# Second Annual Convention of the American Dermatological Association

**Published:** 1878-10

**Authors:** 


### SECOND ANNUAL CONVENTION OF THE AMERICAN DERMATOLOGICAL ASSOCIATION.

HELD AT SARATOGA SPRINGS, N. Y., AUGUST 27, 28 AND 29, 1878.

*(Reported for the Chicago Medical Journal and Examiner, by I. E. Atkinson, M. D., of Baltimore, Md.)*

SARATOGA, AUG. 27, 1878.

The American Dermatological Association met at 9:30 o'clock, a. m., at the Grand Union Hotel. Present, Drs. White, President, Wigglesworth, Duhring, Fox, Piffard, Van Harlingen, Heitzmann, Sherwell, Taylor, and Atkinson; and by invitation, Dr. Graham, of Toronto. After a short business meeting, the regular morning session was opened by the president, who briefly addressed the members, referring to the difficulties of classification and nomenclature in the study of dermatology, and the benefits to follow the labors of the committees appointed at the first meeting, to consider these subjects and to collect statistics of skin diseases in this country. The results of the labors of these committees must have an important influence in determining an improvement in the teaching of dermatology. During the year, the members of the association had actively contributed to dermatological literature, as shown by the list appended. Since the last meeting there had occurred the death of one member, Dr. Silas Durkee, of Boston. Dr. I. E. Atkinson, of Baltimore, then read a paper upon "The Pigmentary Syphiloderm." This lesion, whose existence is not generally admitted by writers, was first described by Hardy, in 1835. It has since then been very ably described, by Fournier especially. It consists in the appearance of faintly colored spots situated upon the necks of females almost exclusively (29 times in 30). The spots are connected with each other by narrow bands of similar pigmentation, leaving small islets of normal or preternaturally whitened integument. The symptom is one of the later secondary manifestations. It is accompanied by no subjective symptoms. Neither is there any elevation of the skin, nor desquamation, nor preceding hyperæmia. Treatment has heretofore had no influence upon the lesion, which is insignificant and must often escape detection, and when seen, upon superficial examination appears more like a soiled skin than a pathological phenomenon. Dr. Heitzmann did not consider the term pigmentary syphiloderm a proper one; the "macular syphiloderm," he thought, would include all cases of this lesion, where the antecedent hyperæmia was of such transitory character as to escape observation. Dr. Duhring, while in Paris several years ago, had observed in Hardy's clinic cases of "syphilide pigmentaire," but had considered them to be not at all satisfactory, and though on the look out for it had only last year observed a case in a young prostitute, 17 years of age, in the hospital of the Univ. of Pa., which he diagnosticated as the pigmentary syphiloderm as described by Fournier, whose description he considered altogether the best—the lesion was quite different from any that he had ever before encountered. Though strongly believing this patient to be syphilitic, he had been unable to identify other syphilitic symptoms. Dr. Sherwell, had frequently seen similar cases, and considered them ordinary chloasma or as vitiligo. He had never succeeded in curing one. Dr. Fox thought an undeserved prominence had been given the lesion, but that while syphilis always precedes it he thought the true condition was vitiligo. He had already expressed his views in the *American Journal of Medical Science*, for April, 1878. Recently he had seen a mottling all over the body like a macular syphiloderm, which the patient, though syphilitic, claimed to have been present since childhood. Dr. Piffard had never recognized the lesion as an independent one. He thought probably two affections were confounded. Vitiligo may be present in syphilitics as well as non-syphilitics. He objected to the definition of vitiligo as given by most writers. Pigment sometimes returns spontaneously to the patches. Dr. Taylor, in a large number of cases, had frequently seen a pigmentary eruption upon the neck, but had observed no points of difference between syphilitics and non-syphilitics. He thought the Latin races more predisposed to pigmentary abnormalities and that this lesion is probably more frequently observed in them than with us. He referred to a physician whose hands were the seat of vitiligoinous patches every summer. The president considered that for the present the burden of proof rested upon those who maintained the independence of the lesion and that more extended observation was required.

Dr. L. A. Duhring then read a paper upon " A case of the so-called Xeroderma of Hebra." The patient was a young woman 17 years old, of Irish parentage, father living, mother dead of cancer, several brothers and sisters alive and healthy, no family history of consumption. This child, at the age of six months began to show upon her nose and cheeks freckles, which increased in number from year to year, and at the age of nine years were as extensive as at present. It was stated that three years ago, while employed at work with machinery, a dozen or more pinhead sized pimples came upon her cheeks and when disappearing left pock-like marks. Dr. Duhring thought this latter an accidental eruption and that the patient was probably mistaken as to their course. Within several years freckles had disappeared from the backs of the hands, leaving atrophy. Almost the whole general surface was pervaded with lentigines, telangiectases and spots of atrophy. The lentigines were scattered at haphazard, sometimes clumped. They were pinhead, but sometimes extended in almost a sheet. In color they were dirty yellow, brown or almost black. Telangiectases were scattered about, but were most numerous upon the neck and chest; they were small and ill-defined, sometimes angular or rounded, and were generally of pinhead size. They were generally inconspicuous, seldom elevated. The skin could be readily picked up. The scalp was invaded. The atrophy upon face and back of hands was noticeable, but not very conspicuous. The patient's general health was good. There were no subjective symptoms. The points of interest were, the early age of the patient when the disease began (as is usual), the slow evolution and chronic course. The atrophy was very superficial. The pigment change was the first in the train of symptoms. The telangiectasis and freckles finally disappear, to be followed by atrophy. Dr. Duhring thought the term Xeroderma inappropriate for the phases of the disease under his observation. The discussion of this paper was postponed until after the reading of the paper by Dr. Taylor, upon the same subject. Dr. George H. Fox, of New York, then read a paper "On the proper use of the term Acne." Dr. Fox argued that the term should be restricted to inflammatory conditions of the sebaceous glands, due to disordered vascular supply arising from internal disorder. Comedo, milium, etc., he regarded as independent disorders. The term should not be used indiscriminately.

Dr. Wigglesworth, of Boston, objected to the term Acne Rosacea, where the sebaceous glands do become inflamed after long capillary congestion. But acne, in other respects, represents a pathological series, of which seborrhœa and comedo are phases.

Dr. Van Harlingen, of Philadelphia, agreed with Dr. Fox as far as concerns acne arising from internal medications, and would like to have these matters settled by a definite terminology. Comedo, he thought, had no influence upon acne, and may be independent of it. Dr. Graham, of Toronto, upon invitation, stated that he had seen cases of sycosis which he regarded as acne. Dr. Sherwell agreed that all forms should be included under the term except those arising from internal artificially-induced conditions. Dr. Piffard thought that in cases where the rosacea exists without any indication of the glands being involved, the term acne should be discarded. There are also cases where rosacea is present with acne. Dr. Fox declared that no cases of acne will occur in healthy people, and that the lesion always arises from internal causes. There is a difference between acne and rosacea, the former representing an active, the latter a passive process. A paper entitled, "A case of scleroderma, by Dr. F. P. Foster, was then read, in Dr. Foster's absence, by Dr. Taylor. A young lady with no family history of cancer, noticed an abrasion upon the nipple. It remained tender for some weeks, and an enlargement the size of a small chestnut appeared at the inner border of the gland. The breast was larger and warmer than natural; there was infiltration, but no appearance of schirrus. Gradually the parts became firmer and involved the skin. Two eminent surgeons diagnosticated schirrus. She consulted Dr.

Foster in February, 1877. The skin of both breasts was then involved, as well as a portion of the left upper extremity. She was subjected to a tonic and a galvanic treatment. Dr. Keyes concurred with Dr. Foster in the diagnosis of scleroderma. The hardness of the skin varied in degree considerably. There was a sense of constriction and shortness of breath. The entire upper half of the trunk became involved, the infiltration extending upon neck and down to left groin. Hyperæsthesia finally made thorough examination impossible. Towards fatal termination there was an irregular fungating mass in the region of the left nipple and ulcers near the navel. The infilitration involved both upper extremities. Towards the last the patient's distress was so great that as much as thirty-six (36) grains of morphia had to be daily hypodermically inserted. Dr. Heitzman thought that the author of the paper had described a case of cancer *en cuirasse*, and not a case of scleroderma. Dr. Van Harlingen did not recognize the symptoms of scleroderma in Dr. Foster's case. Dr. Taylor thought that the presence of ulceration in the case récited should not necessarily exclude a diagnosis of scleroderma, since he had seen ulceration in scleroderma to such an extent that amputation of the leg had to be performed. The president thought the case unlike scleroderma. Dr. Piffard referred to the use of hydrocotyle asiatica, mentioned by Dr. Foster, as used in the treatment. He had used it in lupus, eczema and psoriasis. Had seen lupus improve under its use, but that epididymitis had been occasioned by it.

End of morning session.

### August 27—Afternoon Session.

Dr. Heitzmann then read a paper upon "Epithelium and its Performances," of which the following is an abstract:

If we watch a single living protoplasmic body, for instance, an amœba, a colorless blood-corpuscle, a pus-corpuscle, with high magnifying powers of the microscope (800-1,200) we invariably see a delicate network both within the nucleus and the protoplasm. The body is surrounded by an extremely thin, shining, homogeneous layer, and such a layer always lines vacuoles also, which temporarily or permanently may form in a creeping protoplasmic body. The network of the nucleus, its surrounding shell, the network of the protoplasm and the covering and lining shells both of the body and its vacuoles are formed by the living matter, the active contraction and passive extension of which cause all changes of shape and locomotion during the life of the protoplasm.

All formations in a highly-developed animal body, being analogous to the outer covering layer of a single protoplasmic corpuscle, therefore covering the outer surface and lining all cavities within the body, which are in direct or indirect connection with the outer surface, are termed *epithelia.* Formations on the contrary analogous to the wall of a closed vacuole of a single protoplasmic body, bear the name of *endothelia.* Epithelia are present on the outer surface of the body, the skin and its prolongations, the hairs, nails, sebaceous, sudoriparous and mammary glands; on the cavity termed the intestional tract and its prolongations, mucous and salivary, pepsin, and intestinal glands and the liver ; on the cavity of the respiratory tract and its mucous glands ; on the cavity of the genito-urinary tract, inclusive of all of its prolongations into the kidneys, and the genital glands. Endothelia line the closed cavities of the skull and the spine, all its covering membranes, and all ventricles in the brain and their elongations into the spinal cord ; the cavities of the chest, both pleural and pericardiac ; the cavity of the peritoneum ; all articulations ; all blood and lymph-vessels, inclusive of the cavities of the heart. A thorough distinction between epithelia and endothelia, however, cannot be maintained, as there is a direct communication between both, on the openings of the uterine tubes into the peritoneal cavity ; the epithelial formations of the ovaries are in no communication with the outer world, and the crystalline lens, a formation completely epithelial in nature, is covered by the endothelium of the interior and posterior chambers. Epithelia and endothelia are fully identical in this intimate structure ; there exist single epithelial layers in the body, for instance, those of the bile-ducts, the uriniferous tubules ; and also ciliated endothelia, for instance in the ventricles of the brain and the central canal of the spinal cord.

Epithelia and endothelia represent continuous layers of living matter. The former are the earliest formations in a developing body, after the stage of indifference, started by the segmentation of the ovum, is passed ; they form the epiblast and the hypoblast. These are invariably devoid of blood vessels and lymphatics, while all formations of the mesoblast, inclusive of its upper layer, from which arise the central nervous organs, are provided with blood vessels and lymphatics. The epithelial and endothelial layers are built up by single, polyhedral, protoplasmic bodies, the formerly so-called "epithelial cells." Each body is separated from its neighbors by a narrow cloak of a lifeless, horny cement substance, this being kindred to the basis-substance in the connective tissues. Under the microscope, we can see only the lateral parts of the cloak, which appear in the shape of a pale seam around each epithelium. The network of the living matter within the protoplasm of the epithelium, sends delicate conical offshoots through all formations of the cement substance, both in epi- and endothelia. These offshoots, up to the present time, have been termed "Thorns of Max Schultze," in honor of their discoverer (1864). That the thorns are universal formations in the cement substance, and especially formations of the living matter, thus building the bridges by which all epithelia are uninterruptedly connected, can be proved by different chemical reagents, and by the study of pathological occurrences within the cement substance, viz.: inflammation, fatty degeneration, etc. In the cement substance run the finest terminating fibers of the nerves, also in connection with the thorns, and indirectly with the net work in the interior of the protoplasm.

We distinguish mainly three varieties of epithelia: The flat; the cuboidal, and the columnar or cylindrical. Flat epithelia invariably construct the outer layers in stratified formations ; cuboidal the middle layers, and columnar the lowest layer, nearest to the connective tissue. Single epithelial layers may exhibit any of the named shapes ; in the uriniferous tubules, for instance, we find all the three varieties, according to the calibers of the tubules. Columnar epithelia have two subvarieties, viz., ciliated epithelium, with whip-like elongations on the outer surface, therefore occurring only in single layers. The cilia here are in connection with the network in the interior of the protoplasm (Th. Eimer and E. Klein).

Another variety is the bacillated epithelium, where the outer surface of the cement substance is provided with numerous delicate rods, such as in the intestinal canal and the bile ducts.

All organs of the body termed "glands," are formations of the epithelium. We distinguish two varieties of glands, viz., the acinous and the tubular. A roundish elongation of the epithelium into the connective tissue forms a simple acinous gland, represented by the mucous glands of the oral cavity, the larynx, the trachea. Repeated folding up of the pouch, leads to formation of compound acinous or racemose glands, represented by the sebaceous, the salivary, the lacteal, the prostatic and other mucous glands. A prolongation of the epithelium, prevailing in the longtitudinal direction, is termed a simple, tubular gland, represented by the pepsin and the intestinal glands. Repeated ramifications of the tubules result in the formation of compound tubular glands, represented by the seminiferous and uriniferous tubules. Another sub-variety of compound tubular glands may originate by coiling of the tubule, as we see in the sweat and ceruminal glands.

The main performance of epithelium, besides the protection of the whole body, conduction of terminal nerve-fibers, therefore of sensation, etc., is the elimination of used up material from the body, viz., secretion. Every glandular formation is epithelial, and every epithelial body can be considered as a gland, inasmuch as the secretion is based upon a function of single epithelia.

There are mainly three varieties of secretion, viz., the watery, the mucous and the fatty. The watery secretion cannot directly be studied under the microscope; we only conclude by watching amœbæ fed with carmine particles, that any time, when through the visible contraction of the living matter, without the protoplasm, carmine particles are thrown out from the amœba, a certain amount of its fluid is discharged also.

A liquid, being present one time in the blood, necessarily must pass through the walls of the blood vessels and enter first the protoplasm of the epithelia, before it can be expelled from these, evidently owing to the contraction of the living network of the protoplasm. The watery secretion is performed by the lachyrmal and the sweat glands; the latter produce a fluid greatly varying in the amount of its solid contents and its consistence at different times. Toward approaching death of the body, the perspiration is inspissated and almost mucous in character. The inspissation of the fluid pressed out from the blood vessels of the tufts of the kidneys, is evidently the main performance of the uriniferous tubules.

The mucous secretion directly can be observed under the microscope best on minute particles, cut off from the inner surface of the small intestine of a frog, by addition of a very dilute solution of chromic acid or bichromate of potash, pure water being too rapid in its action. First we see swelling of the protoplasm near to the outer or free surface of the ephithelium. Here the covering cement substance is bulging out, and after having reached the uppermost capacity of expansion, it bursts, and a pale globular body jumps forward, the swelled protoplasm, in which no trace of the former structure can be seen. A number of such pale globules coalesce, and form the jelly like mass called mucus. At other times the whole protoplasm swells within the cloak of the cement substance, and after being freed, still shows the netlike structure of the protoplasm, or isolated granules in a lively motion, the broken particles of the former living matter. Salivary and mucous corpuscles arise from such slow action upon the protoplasm. The cloak of the cement substance, partly or totally emptied, and perforated on one end, gives the appearance of a "goblet cell." A variety of the mucous secretion is that of the stomachic juice, of the bile, and of the semen, in the latter fluid being suspended formations of living matter, the spermatozoids, a direct offspring of the epithelia of the testicles. Saliva represents an intermediate condition between watery and mucous secretion.

The third variety of secretion can best be studied under the microscope on colostrum corpuscles, which are suspended in the serous discharge of the mammary glands for a few days after delivery. Here we see the first formed fat granules still in connection with the network of the living matter within the protoplasm, and we readily arrive at the conclusion, that fat is a directly transformed living matter. During the locomotion of the colostrum corpuscle, very often fat granules are thrown up from its interior (S. Stricker). After a few days, however, no colostrum corpuscles are secreted any more, because the living matter of the epithelia is completely transformed into fat granules, leading to a destruction of the epithelia, the granules of which commingle with a serous fluid, and form the emulsion called milk. This process of fatty change of the living matter of the epithelia of the mammary glands is a remarkably rapid one; on microscopic specimens of the breast in full lactation, we find but little protoplasm unchanged; its greatest part is transformed into fat granules, which having been extracted from the specimen with oil of cloves, leave only the shells of the cement substance behind. The highest degree of fatty change of the protoplasm is reached in the sebaceous and the ceruminal glands.

This paper was discussed by Drs. Atkinson, Fox and Piffard, the latter re-asserting his previously expressed view of the derivation of the horny layer of the epidermis from the stratum lucidum of Oehl, and claimed that the study of dermatology was not exclusively to be conducted with the microscope; that the first object of dermatologists was the cure of disease, and that the microscope is not always our safest guide in diagnosis, and that it is impossible with it alone to distinguish several diseases whose etiology is different, and whose clinical symptoms are very unlike. Dr. Heitzmann replied to Dr. Piffard's objections. The president, Dr. White, then read the report of the committee upon statistics. The committee had succeeded in bringing together, from different districts, nearly 17,000 cases of skin diseases never before reported. The report included six districts, as follows: Boston, New York, Philadelphia, Baltimore, St. Louis and Chicago, giving a total of 13,328 dispensary and 3,035 private cases. Tables of comparative prevalence of the various skin diseases were also read. There was also submitted a report upon leprosy as occurring in this country. The cases were reported from Tracadie, in New Brunswick, in the French settlement, where it has prevailed since the last century. Cases were also reported from Boston, New York, Philadelphia, Baltimore, and a series of sixteen cases observed by Dr. Geddings in Charleston. Reference was also made to cases among the Swedish settlers of the Northwest, and to a possible colony in Minnesota (reported to the chairman by Dr. Hyde). The prevalence of the disease among the Chinese in California was referred to. Most of the cases were of foreign birth, so that the disease may be almost said not to occur with us.

End of first day's session.

AUGUST 28—9.30 A. M.

At the business meeting, Drs. E. B. Bronson, of New York, F. B. Greenough, of Boston, and G. H. Rohé, of Baltimore, were elected active members of the Association. The Nominating committee having reported, the following officers were elected for the ensuing year: President, Dr. L. A. Duhring, of Philadelphia; Vice-Presidents, Dr. J. Nevins Hyde, of Chicago, and Dr. S. Sherwell, of Brooklyn; Secretary, Dr. R. W. Taylor, of New York; Treasurer, Dr. I. E. Atkinson, of Baltimore.

AUGUST 28—10 A. M. REGULAR SESSION.

Dr. L. A. Duhring, of Philadelphia, read a paper upon "A Case of Inflammatory Fungoid Neoplasm." The patient was a married woman, 58 years old, the mother of four children, and weighing 174 lbs. Health had always been good, and catamenia, which ceased at 46 years, regular. She has had suppression of urine occasionally, and some anasarca quite often. Her mother died of cancer of the face. In August, 1876, she experienced after a cold bath, soreness and pain. Two days afterward her skin became red, and three hours afterwards wheals appeared which lasted 24 hours. Since then has had various eruptions of urticaria and eczema. Upon 10th October, a lesion, which is still present, came upon her forehead, suddenly, as a red spot, like a superficial burn; it grew, showing many variations of color. In August, 1877, it began to rise slowly, like a boil, but without subjective symptoms. In one day, nine new small tumors came upon the scalp; they were like gristle to the touch, freely moveable, and soon disappeared. Near the original patch, in September, 1877, numerous small tumors came, without subjective symptoms. In following June, also, similar tumors came suddenly upon abdomen. Later, sensitive tumors appeared upon the groins; also, there appeared, without her knowing when, tumors upon her buttocks and back. Dr. Duhring saw her first October 25th, 1877. The previous history, as given by patient, Dr. D. thinks reliable, she being a remarkably intelligent woman. When seen by Dr. D., the lesion involving the right side of the forehead (the original lesion), was of size of a half cherry, of a raspberry color, tense, containing no pus, tuberculated and furrowed. Inside of right arm was an oval papule, dull red in color. The natural lines of the skin were exaggerated. There was another tumor in the posterior fold of right axilla. Several patches were upon the abdomen. There was a patch in the groin half an inch in height, shape of a hen's egg, and smooth. There was much dusty yellow pigmentation. On October 26th, Dr. Duhring observed a new lesion that had appeared since the night before. It was near the nipple, olive shaped, rough, of a deep, pinkish color, soft and supple—not inflammatory, not changing color to pressure. It was evident that while tumors were continually appearing, they were almost as rapidly disappearing. Some were persistent, as those upon forehead. Some lesions grew with great rapidity. The growths extended over forehead, eyebrow and scalp, both flat and tuberculated. Iodide of potash and arsenic unquestionably aggravated the disease. Some of the patches disappeared as rapidly as urticaria. One tumor upon the left cheek softened and suppurated. This tumor, after it had existed three months, was excised. Its cut surface was grayish yellow, and firm, like sarcoma. Its weight was 1 oz. The principal features of the microscopic section of this tumor, which was exhibited, was that of inflammatory round cell infiltration. The points of the case were the variety of the lesions, their number and sudden appearance and rapid disappearance, and the preservation of the general health. It was a true skin disease. The patches were of different sizes, from split-pea to size of egg, sometimes fungoid, soft or firm, lobulated and furrowed, even in the flat portions, smooth or rough, crusted or not, without symmetry. All portions of the body were invaded. The only subjective symptom was variable itching. For some time, Dr. D. was unable to name the disease, or to find record of an analogous case. Hebra has, however, described a case. Hans Hebra has also described a case. Geber has also written an account of Hebra's case. Dr. Duhring exhibited the case to the members. The patient pointed out several large tumors upon different portions of the body, which had appeared in the few weeks interval of Dr. Duhring's absence from Philadelphia. Some of these were quite as large as pigeon's eggs, and were already undergoing involution, the process beginning in the center. A remarkable feature was the perfectly normal condition of the skin at the points where tumors had been, but had disappeared. There was not the slightest evidence of interstitial absorption. Photographs and drawings were also exhibited. Dr. Duhring regarded the affection as benignant. He thought the fluid extract of ergot had had a decidedly beneficial influence upon the growths. Dr. Piffard had seen exhibited at the N. Y. Dermatological Society two somewhat similar cases. Dr. Fox thought that milder cases of a somewhat similar affection were not seldom seen. Dr. Sherwell described a case which he thought somewhat similar, and which proved fatal. A paper by Dr. W. A. Hardaway, of St. Louis, entitled, "Treatment of Hirsuties," was then read, in the absence of Dr. Hardaway, by Dr. Taylor. In the treatment of hirsuties, the essential point being the destruction of the hair follicles, it is evident that ordinary epilation only stimulates the hair growth. To destroy the papilla, he employs a method first used by Dr. Michel for the treatment of ingrowing eyelashes—that of electrolysis. He uses by preference a Hill battery of from 8 to 20 cells, the needle, if fixed in an electrode made of a hollow bone pencil-holder—the needle-holder being attached to the negative pole—a wet sponge to the positive pole. The needle is to be inserted along the hair as far as the papillæ, and the current stopped when a slight white frothing is observed at the orifice. The hair may then be removed by epilation, and unless it comes easily, the operation is a failure. With eight elements, two to five seconds are sufficient; with twenty elements, the current is continued not more than one second. With the instrument exhibited, he was enabled to destroy 160 hairs in 30 minutes. No scar resulted. There is some pain in the operation. The needle should be the smallest cambric. Dr. H. has also occasionally employed a solution of were soft and impressible. Reflecting on this state of things, and believing my patient in too prostrated a condition for the manipulation necessary to restore the fundus, I was led to hope also, that if a little tone could be imparted to the fibers of the body and fundus, the discharge would diminish and possibly a spontaneous restoration result. Continuing the former supporting treatment and quietude, I administered half a drachm of Squibb's fluid extract ergot every four hours. From the time the ergot was commenced the discharge gradually became less, the fundus and body firmer, and the os smaller until the 28th of February, 1878, when the uterus was restored to its proper size, shape and position. The os was closed, the sound entered the uterine cavity three inches, and penetrated three inches into the uterine cavity by deep pressure above the symphysis, while two fingers pushed the uterus upward, the natural outlines of the fundus could be distinguished. Tonics and a nutritious diet were continued from this time forward, and were followed by uninterrupted improvement, until the patient was entirely cured.

My father, Dr. Wm. H. Byford, saw the patient with me February 10th. He fully verified my diagnosis, and coincided with me in the treatment. He also examined the patient August 16th, 1878, and assured me that the uterus was not only restored to its natural position, but was in a healthy condition.

I will not presume to make any comments on this case, as the points will readily present themselves to the reader. The only thing to which I will draw attention is the steady improvement following the administration of ergot. It is true, nature might have done all that was necessary for the restoration of the uterus without this remedy ; it certainly produced no injurious effects.

In this connection it may be apropos to mention a case which was under the observation of my father where spontaneous restoration took place after the uterus had been completely inverted for fifteen years.

Henry T. Byford, M. D.

*Unusually High Temperature in a Case of Sunstroke.*

At 3 p, m. on the 17th day of July I was called to see P. H——, a brick-yard employé. I found the man entirely unconscious, face flushed, breathing laboriously, with now and then slight convulsive contraction of the muscles of mastication, and skin dry and excessively hot—so hot that to touch him was unpleasant. On inquiry I ascertained that he had not been feeling very well all day, and about half an hour before my arrival fell down unconscious, and had been carried under the shed where I found him.

Astonished at the great heat of his skin, I determined to ascertain his temperature before carrying out the treatment I had concluded upon. While removing his clothes to get at the axilla, etc., I despatched some bystanders for a large cake of ice. I placed the thermometer in his axilla—first noticing that the mercury stood at 95°—and drew his arm firmly against the side of the body. The mercury began to rise rapidly, and in less than a minute reached the very top of the tube in the thermometer. Of course I was amazed, but there was no doubting the fact of the register. My thermometer is of the best English make, and has been proven trustworthy. It is scaled to 112°, the tube reaches a distance equal to 1° above, so that the case had a temperature marked to 113°. The rapidity with which the mercury rose to this height convinces me that the register would have been higher had there been more space for its markings. I entertained very little hope of the man's recovery. While waiting for the ice, I uncovered his body of clothing and dashed cold water all over him repeatedly, with, at first, scarcely any response from his nervous system. Soon, however, there followed a deeper and more complete respiration after each application of water. When the ice arrived I placed large pieces over the groins, in both axillae, to the sides of the neck, and embanked the head in small fragments, covered the abdomen, and in fact put it everywhere that it could be made to hold, choosing by preference such parts of the body where large blood-vessels come near to the external surface. By this means—ice and cold water—in an hour's time the temperature was reduced to $99\frac{1}{2}$°. As the temperature came to 100° the man began to show more searches into the mucorini. This plan is as follows: A glass ring four to five mm. in height, with an interior diameter of about fifteen mm., is fastened to a glass slide with Canada balsam. The spore to be cultivated is then sown in an appropriate nutritive fluid upon a very thin cover glass, the diameter of which is equal to that of the glass ring. The cell is then completed by laying the cover glass with the nutrient drop underneath, upon the glass ring; a few minute drops of oil upon the surface of the latter, upon which the cover glass rests, serves to protect the cell from external influences. All fluids and appliances must be as pure as it is possible to make them. This method may be called the "cell culture." In a cell like this a very small portion of hair shaft drawn from the follicle is sown, the nutritive fluid being either orange juice, Pastein's fluid, with or without sugar, decoction of horsedung, or any other approved fluid. The first two being preferable. In a large majority of cells thus planted there will be no change, some will develop bacteria, or moulds of adventitious nature. In a few cells a mode of development will take place, which is characteristic of the fungus of trichophyton, and can readily be distinguished from foreign fungus, the latter either thrusting its hyphæ under the cover-glass or developing within the cell from a single center, and readily discoverable, the mass of trichophyton spores remaining entirely quiescent. Where, however, the cultivation is a successful one, there is a simultaneous, uniform, multitudinous growth of hyphæ, accompanied sometimes by a decided swelling of the spores, which may become many times their original size; [figure shown] the latter, however, ceases as soon as hyphæ become developed extensively. These hyphæ, where the nutrient fluid is abundant, tend to form a closely meshed mycelium, and to form but few reproductive organs. Where, however, the nutrient fluid is soon used up, reproductive organs are freely developed. In all instances, without exception, these organs have been similar to those of the mucors, sporangial. In these successful cultures, adventitious fungous growths have never occurred. The fungus then, of trichophytina, is identical with mucor mucedo. The method is easily practiced, and only requires care, attention and perseverance, in spite of the many disappointments arising from the tendancy of the spores to remain quiescent.

Dr. Piffard objected to the process of examination as not being free from sources of fallacy.

The next paper was upon a "Case of Ulcerative Scrofuloderm," by Dr. Arthur Van Harlingen, of Philadelphia. The case was of an elderly woman, who first developed symptoms akin to eczema, but rapidly became subject to ulcerations of the general surface of a very peculiar character. Her strength gradually failed, but before death there was developed in the tongue and on the hard palate, nodules. Dr. Van Harlingen was somewhat inclined to attribute the case to a tuberculosis of the skin, but adopted the title given above. The result was fatal. Dr. Duhring, on seeing the case, was at first disposed to think it an old eczema, but the depth of the ulceration changed his mind. When he again saw the patient after a considerable interval, he was much astonished, and felt unable to decide about it. He had never seen anything like it before. He agreed with Dr. Van Harlinger as to its nature.

### August 29—10 a. m.

Dr. R. W. Taylor, of New York, presented a paper entitled, "A Further Contribution to the Study of the Xeroderma of Hebra." This paper is supplementary to a paper read at the first annual meeting of the Association at Niagara Falls, Sept., 1877, a synopsis of which has already been given. Dr. Taylor has since had three of the cases under close observation, and has devoted much study to them. He was now able to report his conclusions. The lesions of this rare affection are vast numbers of pigmented spots, upon the face, neck, back of hands, fore-arms, breasts, and partly legs, also teleangiectases, atrophies, and new growths. The spots come and go. The course is as follows: First, the vessel becomes telangiectatic, may be in from 24 to 48 hours; the brown spots come upon the site of this dilatation, which may be linear or of various shapes; brown spots may appear, however, without the preceding vascular enlargement. The telangiectases may again come in the same spot, atrophy gradually taking place with gradual disappearance of pigment. The essence of the disease is vascular new formation —as epiphenomena are neoplastic formations, which he thought not necessarily malignant. The tumors which are prone to occur upon the face, may become withered, were twisted off with some hæmorrhage, and healed slowly, he thought them not epitheliomatous. The activity of the disease ends in early life, but the pigmentation and atrophy are permanent. Xeroderma is a bad name, since until atrophy occurs there is no dryness. It is a vascular neoplasm, and Dr. Taylor proposed as the name for it, angioma pigmentosum et atrophicum.

Dr. Heitzmann stated that he had been the first to observe these cases. He had now a patient in whom the disease was of 20 years duration. A tumor which he regarded, had appeared, and which he had removed five times. The specimen exhibited by Dr. Taylor, from one of the tumors of the face, Dr. Hertzmann declared to be myxomatous, and would call the tumor myxo-angioma. He accepted Dr. Taylor's definition of the disease. Dr. Duhring was unable to determine Dr. Taylor's succession of symptoms in his own case. He was pretty sure that freckles first appeared exactly like lentigo. In places there were no vascular dilatations at all. He had not seen the pigmentary lesion follow telangiectasis. Dr. Taylor thought Dr. Duhring's case was too old to contradict previous vascular dilatation, as the latter had time to disappear, leaving lentigines and atrophy. Dr. Piffard thought the specimen one of malignant neoplasm, a myxo-carcinomatous growth.

The President then presented the report of the committee upon Nomenclature.

The following being recommended :

Class I. Disorders of the Glands.

1 Of the Sweat Glands.
Hyperidrosis.
Miliaria crystallina.
Anidrosis.
Bromidrosis.
Chromidrosis.

2 Of the Sebaceous Glands.
Seborrhœa, (a) S. oleosa, (b) S. sicca.
Comedo.
Cyst, (a) milium, (b) wen.
Molluscum sebaceum.
Diminished secretion.

Class II. Inflammations.

Exanthemata contagiosa.
Erythema simplex.
Erythema multiforme. (a) E. papulatum, (b) E. bullosum. (c) E. nodosum.

Urticaria.
Dermatitis.
(a) D. traumatica. (b) D. venenata. (c) D. calorica.*
Erysipelas.
Furuncle.
Anthrax.
Phlegmona diffusa.
Pustula maligna.
Herpes. (a) H. facialis. (b) H. progenitalis.
H. zoster.
Psoriasis.
Pityriasis rubra.
Lichen. (a) L. planus. (b) L. ruber.
Eczema. (a) E. erythematosum. (b) E. papulatum. (c) E. vesiculosum. (d) E. madidans. (e) E. pustulosum. (f) E. rubrum. (g) E. squamosum.
Prurigo.
Acne.
Impetigo.
I. Contagiosa.
I. Herpetiformis.
Ecthyma.
Pemphigus.

Class III. Hæmorrhages.

Purpura. (a) P. simplex. (b) P. hæmorrhagica.

Class IV. Hypertrophies.
1 Of Pigment.
Lentigo.
Chloasma. (a) C. locale. (b) C. universale.
2 Of Epidermal and Pappillary Layers.
Keratosis.
Callositas.
Cornu cutaneum.
Verruca.
Verruca necrogenica.
Xerosis.
Icthyosis.
Hypertrophy of nail.
3 Hirsuties.
4 Of Connective Tissue.
Scleroderma.
Sclerema neonatorum.
Morphœa.
Elephantiasis Arabum.
Rosacca. (a) R. erythem. (b) R. hypertroph.
Framboesia.

Class V. Atrophies.
1 Of Pigment.
Leucoderma.
Albinismus.
Vitiligo.
Canities.
2 Of Hair.
Alopecia.
Alopecia areata.
Alopecia furfuracea.
Atrophia pilorum propria.
3 Of Nail.
4 Of Cutis.
Atrophia senilis.
Atrophia maculosa et striata.

Class VI. New Growths (benign).
1 Of Connective Tissue.
Keloid.
Cicatrix.
Fibroma.
Neuroma.
Xanthoma.
2 Of Vessels.
Angioma.
Angioma pigmentosum et atrophicum.
Angioma cavernosum.
Lymphangioma.
3 Of Granulation Tissue.
Rhino-scleroma.
Lupus erythematosus.

* These indicating affections not properly included in other titles of this class.

Lupus vulgaris.
Scrofuloderma.
Syphiloderma.
(a) S. erythem. (b) S. papul.
(c) S. pust. (d) S. tubercul.
(e) S. gummosum.
Lepra. (a) L. tuberculatum.
(b) L. maculatum· (c) L. anæstheticum.
Carcinoma.
Sarcoma.

Class VII. Ulcers.

Class VIII. Neuroses.
Hyperæsthesia. (a) Pruritus.
(b) Dermatalgia.
Anæsthesia

Class IX. Parasitic Affections.
1 VEGETABLE PARASITES
Tinea favosa.
Tinea trichophytina. (a) T. circinata. (b) T. tonsurans. (c) T. sycosis.
Tinea vesicolor.

2 ANIMAL PARASITES.
Scabies.
Pediculosis capillitii.
Pediculosis corporis.
Pediculosis pubis.

This report, after discussion, was adopted, the ayes and nays being called for by Dr. Piffard. The ayes being Drs. Taylor, Wigglesworth, Duhring, Van Harlingen, Heitzmann and Atkinson; the nays being Drs. Piffard, Fox and Sherwell. The retiring president then expressed his gratitude for the consideration shown. He believed that the success of the association had been assured by the high order of the papers presented before it, and predicated a prosperous future for American dermatology. He read by title his paper upon "A Case of Recurrent Cutaneous Hæmorrhage with Urticarial and Bullous Efflorescences." The new president, Dr. Duhring, upon assuming the chair, promised to do all in his power to promote the interests of the association, and bespoke the co-operation of the members. He indulged the hope that the ensuing year will call forth scientific research in dermatology, and still further cement the friendships of the members of the association.

The association then adjourned to meet in the city of New York upon the first Tuesday of September, 1879.

---

A NEW DISEASE.—According to reports from Readville, Mass., several of the operatives in a curled hair factory have died of a disease supposed to have been introduced with the hair of diseased animals, principally Siberian horses.

